# EHMTI-0061. Do the interictal microembolic signals have a role in higher cortical dysfunctions during migraine aura?

**DOI:** 10.1186/1129-2377-15-S1-E27

**Published:** 2014-09-18

**Authors:** A Podgorac, I Petrusic, J Zidverc-Trajkovic, A Radojicic, Z Jovanovic, N Covickovic-Sternic

**Affiliations:** 1 School of Medicine, University of Belgrade, Belgrade, Serbia; 2 Headache Center, Neurology Clinic CCS, Belgrade, Serbia; 3 Department for Cerebrovascular disorders and Headaches, Neurology Clinic CCS, Belgrade, Serbia

## Introduction

Higher cortical functions (HCF) impairment during aura in migraine patients is more frequent than previously thought.

The aim of this study was to evaluate prevalence and clinical impact of interictal microembolic signals (MES) in migraine patients with HCF disturbances during aura.

## Method

This study was carried out on 68 patients (34 migraineurs with higher cortical functions (HCF) disturbances during the aura (HCD group) and 34 migraineurs with only visual or visual and sensory symptoms (Control group I) and 31 healthy controls (Control group II). Demographic data, disease features and detected MES between these groups were analyzed. Furthermore, patients with HCF disturbances were analyzed according to presence of MES.

## Results

There was no statistically significant difference in terms of gender, age at the time of examination and age at the time of the onset of disease between three groups. Aura was longer (34.71 ± 18.05 vs. 23.87 ± 13.64, p=0.002) and frequency of aura per year was higher (16.29 ± 14.21 vs. 10.10 ± 11.00, p=0.029) in the HCD group as compared to the Control group I. Also, sensory aura was significantly more present in HCD group (p<0.001). MES were detected in 10 (29.4%) patients from HCD group, which was significantly higher compared to 1 (3.2%) in Control group I and 2 (5.9%) in Control group II (χ2=7,909, p=0.005; χ2=6,476, p=0.011).

## Conclusion

High prevalence and frequency is detected in migraine patients with HCF disturbances during aura. The exact pathophysiological mechanism of this finding is not clear and requires additional investigations.

No conflict of interest.

